# Coronary artery stenosis-related perfusion ratio using dynamic computed tomography myocardial perfusion imaging: a pilot for identification of hemodynamically significant coronary artery disease

**DOI:** 10.1007/s12928-019-00627-4

**Published:** 2019-10-19

**Authors:** Natsumi Kuwahara, Yuki Tanabe, Teruhito Kido, Akira Kurata, Teruyoshi Uetani, Hitomi Ochi, Naoto Kawaguchi, Tomoyuki Kido, Shuntaro Ikeda, Osamu Yamaguchi, Migiwa Asano, Teruhito Mochizuki

**Affiliations:** 1 Department of Radiology, Ehime University Graduate School of Medicine, Shitsukawa, Toon, Ehime 791-0295 Japan; 2 Department of Cardiology, Pulmonology, Hypertension and Nephrology, Ehime University Graduate School of Medicine, Shitsukawa, Toon, Ehime 791-0295 Japan; 3 Department of Legal Medicine, Ehime University Graduate School of Medicine, Shitsukawa, Toon, Ehime 791-0295 Japan

**Keywords:** Computed tomography, Coronary artery disease, Myocardial ischemia, Fractional flow reserve, Myocardial blood flow

## Abstract

The purpose of this study was to evaluate the feasibility of the stenosis-related quantitative perfusion ratio (QPR) for detecting hemodynamically significant coronary artery disease (CAD). Twenty-seven patients were retrospectively enrolled. All patients underwent dynamic myocardial computed tomography perfusion (CTP) and coronary computed tomography angiography (CTA) before invasive coronary angiography (ICA) measuring the fractional flow reserve (FFR). Coronary lesions with FFR ≤ 0.8 were defined as hemodynamically significant CAD. The myocardial blood flow (MBF) was calculated using dynamic CTP data, and CT-QPR was calculated as the CT-MBF relative to the reference CT-MBF. The stenosis-related CT-MBF and QPR were calculated using Voronoi diagram-based myocardial segmentation from coronary CTA data. The relationships between FFR and stenosis-related CT-MBF or QPR and the diagnostic performance of the stenosis-related CT-MBF and QPR were evaluated. Of 81 vessels, FFR was measured in 39 vessels, and 20 vessels (51%) in 15 patients were diagnosed as hemodynamically significant CAD. The stenosis-related CT-QPR showed better correlation (*r* = 0.70, *p *< 0.05) than CT-MBF (*r* = 0.56, *p* < 0.05). Sensitivity and specificity for detecting hemodynamically significant CAD were 95% and 58% for CT-MBF, and 95% and 90% for CT-QPR, respectively. The area under the receiver operating characteristic curve for the CT-QPR was significantly higher than that for the CT-MBF (0.94 vs. 0.79; *p* < 0.05). The stenosis-related CT-QPR derived from dynamic myocardial CTP and coronary CTA showed a better correlation with FFR and a higher diagnostic performance for detecting hemodynamically significant CAD than the stenosis-related CT-MBF.

## Introduction

Coronary computed tomography angiography (CTA) is widely used as a non-invasive tool for the assessment of coronary arteries [1, 2]. When assessing coronary artery disease (CAD), it is important to assess the hemodynamic significance of a coronary artery stenosis to determine the optimal treatment strategy prior to revascularization [3]. Fractional flow reserve (FFR) is the gold standard for the identification of hemodynamically significant CAD [4]; however, an invasive approach is required to measure FFR by invasive coronary angiography (ICA). Myocardial computed tomography perfusion (CTP) imaging has emerged as a non-invasive technique for myocardial perfusion imaging [5, 6]. Moreover, dynamic myocardial CTP imaging allows to provide quantitative hemodynamic parameters such as computed tomography-derived myocardial blood flow (CT-MBF) [5–7]. However, CT-MBF varies between individuals even in the normal myocardium [8, 9], and the standardized cutoff has not been established yet. We proposed the computed tomography-derived quantitative perfusion ratio (CT-QPR) as a relative measure of CT-MBF and hypothesized that the CT-QPR would detect hemodynamically significant CAD more effectively than the absolute CT-MBF. Moreover, Voronoi diagram-based myocardial segmentation, which is one of the segmentation methods of myocardial perfusion territory using coronary CTA data, allows for the accurate assessment of stenosis-specific myocardial perfusion independently of variations in the myocardial perfusion territory [10, 11]. The purpose of this study was to evaluate the feasibility of the stenosis-related CT-QPR for detecting hemodynamically significant CAD assessed by the FFR.

## Methods

### Study population

This study was approved as a retrospective observational study by the local institutional review board, and the need for informed consent was waived. Twenty-nine patients who underwent comprehensive cardiac CT prior to ICA with FFR measurement between January 2013 and January 2015 were recruited for this study from the clinical database. The patients were screened for CAD based on effort or resting angina documented by ST-T changes on the electrocardiogram (ECG), reduction of angina symptoms after the administration of nitroglycerin, or multiple coronary risk factors. The attending physician determined the indications for cardiac CT and ICA. The exclusion criteria for the patients were: (1) age < 20 years, (2) coronary artery bypass grafting, (3) acute or old myocardial infarction, (4) left ventricular ejection fraction < 20%, (4) greater than first degree atrioventricular block, (5) complete left bundle branch block, (6) moderate or severe valvular heart disease, (7) cardiomyopathy, and (8) inadequate datasets such as poor image quality of coronary CTA, insufficient CT-MBF analysis, or incomplete FFR measurement. The radiation dose was calculated from the dose-length product with a conversion coefficient (0.014 mSv × mGy^−1^ × cm^−1^).

### Scan protocol of comprehensive cardiac CT

All patients underwent stress dynamic cardiac CT, which consisted of pharmacological stress dynamic CTP and coronary CTA, as previously described [12]. A 256-slice multidetector row CT (Brilliance iCT; Philips Healthcare, Cleveland, OH, USA) and an automatic dual injector (Stellant DualFlow; Nihon Medrad KK, Osaka, Japan) were used. A timing bolus was applied to estimate the scan timing of dynamic CTP and coronary CTA using a 20% solution of the contrast medium iopamidol (370 mg iodine/mL; Bayer Yakuhin, Ltd., Osaka, Japan) diluted with saline (5.0 mL/s for 10 s), followed by a saline chaser (5.0 mL/s for 4 s). Pharmacological stress was induced by intravenous infusion of adenosine triphosphate (ATP, 20 mg, 0.16 mg/kg/min, 5 min; Daiichi Sankyo, Inc., Tokyo, Japan). Three minutes after ATP loading, a series of stress dynamic CTP images were obtained at every heartbeat or once every other heartbeat, using a bolus of non-diluted contrast medium followed by a saline chaser of the same volume and injection rate as the timing bolus. The scan parameters were as follows: prospective ECG-gated dynamic mode targeting a phase of 40% RR interval; tube voltage, 80 or 100 kVp; tube current–time product, 75–110 mAs; tube-rotation-time, 270 ms; detector collimation, 64 × 1.25 mm. Subsequently, 0.6 mg of nitroglycerin was administered to all patients prior to CTA, and 0.125 mg/kg of landiolol hydrochloride (Corebeta; Ono Pharmaceutical Co., Osaka, Japan) was administered when the heart rate 5 min prior to the coronary CTA was > 70 beats/min. Coronary CTA was performed using contrast medium followed by a saline chaser of the same volume and injection rate as the timing bolus. The scan parameters were as follows: prospective or retrospective ECG-gated scan mode for heart rates ≤ 65 or > 65 beats/min targeting a phase for dose modulation of 75% RR interval; pitch factor, 0.14; tube voltage, 120 kVp; tube current–time product, 325.4 ± 207.4 mAs; detector collimation, 2 × 128 × 0.625 mm with a dynamic z-focal spot.

For the coronary CTA assessment, axial images were reconstructed from the cardiac phase with the fewest artifacts using a 0.8-mm slice thickness, a 0.4-mm section intervals reconstruction increment, and a medium cardiac kernel. For CT-MBF quantification, dynamic CTP datasets with 1.25-mm thicknesses were reconstructed using a 360° reconstruction algorithm. Elastic registration for motion compensation and a spatio-diffusion filter for reducing noise spikes over time were applied using commercially available software (IntelliSpace Portal; Philips Healthcare, Cleveland, OH, USA).

### Post-processing and analysis of coronary CTA and dynamic myocardial CTP

The datasets of CTA and dynamic CTP were transferred to a commercially available software (Synapse Vincent ver.5; Fujifilm Medical Systems, Tokyo, Japan). Two experienced radiologists (with 2 and 15 years of experience in cardiac CT, respectively) performed coronary CTA assessment and reconstructed the three-dimensional coronary artery tree. The corresponding myocardial territory for each coronary branch was automatically determined from coronary CTA data using a commercially available software based on the Voronoi diagram as previously reported [13, 14]. Briefly, the Voronoi diagrams were used to allocate each voxel of the left ventricular myocardium to the spatially nearest coronary artery branch as its territory. The vessel with a stenosis (≥ 50% luminal reduction) or un-assessable calcified segments were assigned as significant stenosis in the CTA assessment. When multiple stenoses were seen in the same coronary vessel, the proximal stenosis was considered as the culprit stenosis. The two operators determined by consensus the seed points of a stenosis in the CTA without referring to ICA images or FFR information.

**Fig. 1:**
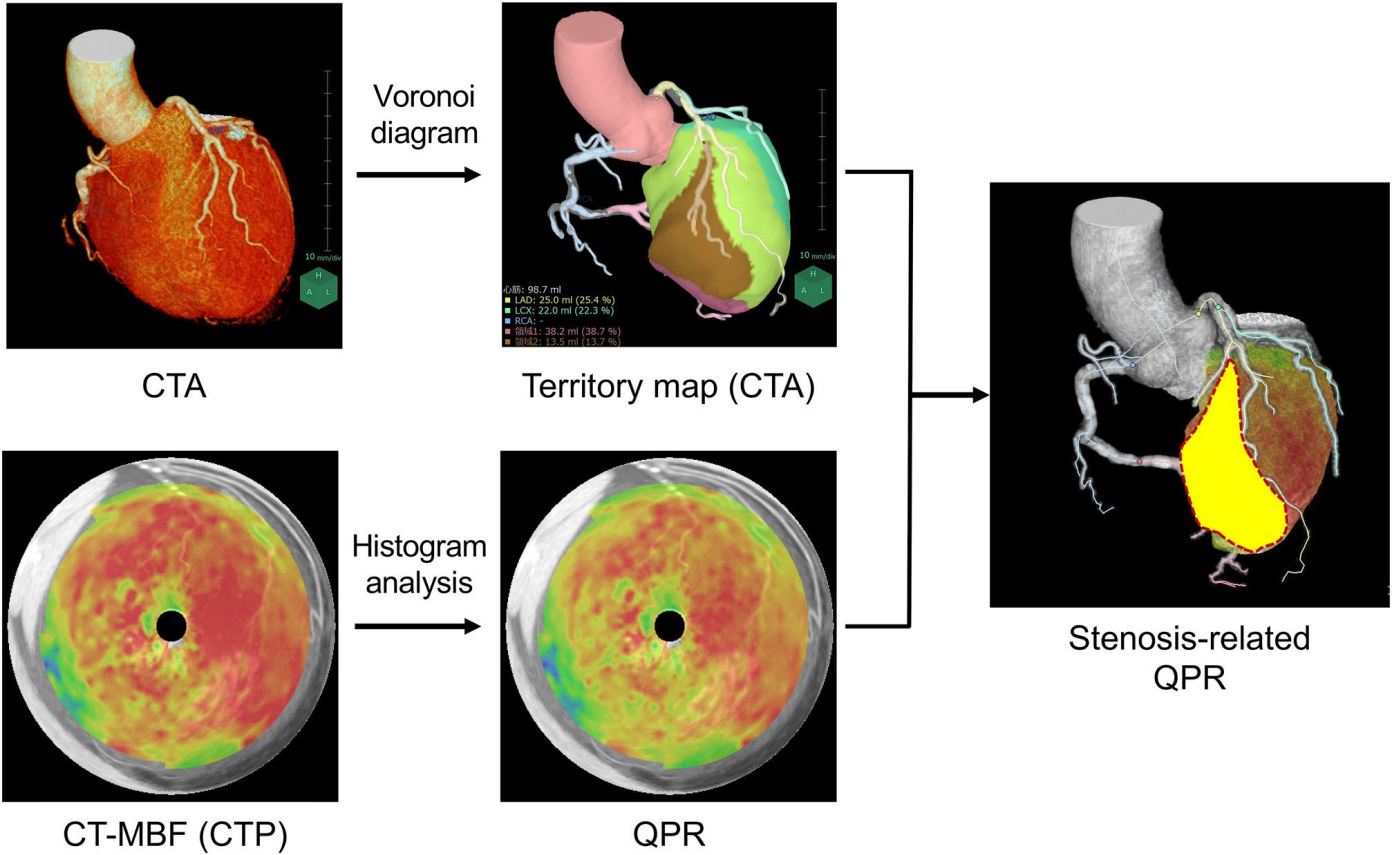
Flow chart for the calculation of the stenosis-related CT-QPR. *CTA* computed tomography angiography, *CT*-*MBF* computed tomography-derived myocardial blood flow, *CTP* computed tomography perfusion, *CT*-*QPR* computed tomography-derived quantitative perfusion ratio

CT-MBF (mL/g/min) for each voxel was calculated at the voxel level using a deconvolution analysis [12]. The reference CT-MBF was automatically calculated by histogram analysis, and CT-QPR was defined as the relative CT-MBF to the reference CT-MBF [13, 14]. The two post-processing datasets of myocardial territories obtained from coronary CTA and CT-MBF, as well as the QPR obtained from dynamic myocardial CTP, were three-dimensionally imposed using non-rigid registration with the integrated software. The stenosis-related CT-MBF and QPR were defined as the regional values in the myocardium perfused by the distal artery from the seed points on the CTA image (Fig. 1). We assessed the diagnostic performance of stenosis-related CT-MBF and QPR for detecting hemodynamically significant CAD assessed by the FFR.

### ICA and FFR analysis

ICA was performed according to the standard procedures using 5-Fr or 6-Fr coronary catheters. Selective coronary angiography was performed in at least six projections for the left coronary artery and three projections for the right coronary artery. The severity of the coronary artery stenosis was assessed using a commercially available quantitative coronary angiography (QCA) software (Pie Medical Imaging BV, Maastricht, The Netherlands). The FFR was measured for vessels with a QCA value between 30 and 69% using pressure-monitoring guidewires (Verrata; Philips North America Corporation, Andover, MA, USA; or Aeris; Abbott Vascular, Temecula, CA, USA) during ICA at the discretion of the attending interventional cardiologists. Hyperemia was induced by intravenous infusion of ATP (0.16 mg/kg/min) as previously described [15]. FFR values ≤ 0.80 were considered to indicate hemodynamically significant CAD [16].

### Statistical analysis

Continuous data were expressed as the mean ± standard deviation or median (25–75 percentiles), as appropriate. The scan heart rates were compared during stress and rest using the paired *t* test. The interobserver agreement was calculated using an interclass correlation coefficient for the stenosis-related CT-MBF and QPR. The coefficient of variation (CV) was calculated as the ratio of the standard deviation to the mean of the CT-MBF and QPR to demonstrate the degree of variability. The correlations between the FFR and stenosis-related CT-MBF, as well as between the FFR and QPR, were evaluated using Spearman’s rank correlation coefficient. Comparisons of stenosis-related CT-MBF and QPR between hemodynamically non-significant and significant CAD were performed using Student’s *t* test. Receiver operating characteristic (ROC) curve analysis was performed to determine the cutoff value of the stenosis-related CT-MBF and QPR to predict hemodynamically significant CAD (FFR ≤ 0.8) using Youden’s index. The sensitivity, specificity, positive predictive value, and negative predictive value for detecting hemodynamically significant CAD (FFR ≤ 0.8) were analyzed using stenosis-related CT-MBF and QPR. The areas under the ROC curves (AUCs) were compared between stenosis-related CT-MBF and QPR using a previously described method [17]. All statistical data are reported with 95% confidence intervals (CI). Probability values with *p *< 0.05 were considered statistically significant. For statistical analyses, we used commercially available software (JMP version 13.0; SAS Institute, Inc., Cary, NC, USA).

## Results

### Study population

A total of 29 patients were extracted from our database. Two patients were excluded because the quality of their CTP images was insufficient; thus, 27 (93%) patients were enrolled as study subjects. Table 1 presents the patients’ characteristics. None of the patients experienced worsening angina or cardiac events during the imaging sessions. All patients successfully underwent whole-heart stress dynamic CTP imaging. The scan heart rate increased significantly from 59.5 ± 8.0 beats/min at rest to 81.8 ± 13.3 beats/min during pharmacological stress (*p* < 0.05). A pre-test probability, based on a 19-year follow-up study of a representative Japanese population [18], determined the 10-year CAD death pre-test probabilities as < 0.5% (*n* = 1), 0.5–1% (*n* = 2), 1–2% (*n* = 5), 2–5% (*n* = 8), 5–10% (*n* = 6), and > 10% (*n* = 5). Prospective and retrospective ECG-gated scans were performed on 14 patients (51.9%) and 13 patients (48.1%), respectively, during coronary CTA. The mean effective radiation doses for CTP and CTA were 10.2 ± 1.3 mSv and 5.4 ± 3.4 mSv, respectively. The mean total radiation dose for CTP and CTA was 15.4 ± 3.8 mSv. The total amount of contrast medium was 96.9 ± 10.2 mL.

**Table 1:** Patient characteristics (*n* = 27)

Age (years)	69.3 ± 8.3
Male	20 (74%)
Height (cm)	160.2 ± 6.9
Weight (kg)	61.6 ± 11.7
Body mass index	23.9 ± 3.6
Coronary risk factors	
Hypertension	17 (62%)
Hyperlipidemia	12 (44%)
Diabetes mellitus	12 (44%)
Smoking	18 (67%)
Family history of coronary artery disease	4 (15%)
Chest pain	8 (30%)
LVEF by echocardiogram	72.1 ± 9.2
CACS	896.4 (264.0–1398.8)
CACS = 0	1 (4%)
CACS = 1–99	3 (11%)
CACS = 100–399	6 (22%)
CACS > 400	17 (63%)

The data presented are the numbers (percentages), the means ± standard deviations, or the medians (25–75 percentiles)

*LVEF* left ventricular ejection fraction, *CACS* coronary artery calcium score

### Characteristics of the coronary vessels assessed using ICA and FFR

QCA was measured for 81 vessels of 27 patients. QCA values below 30% were determined in 39 vessels, 39 vessels showed a QCA between 30 and 69%, and 3 vessels had a QCA of 70% or more. The total of 39 intermediate stenotic vessels (30–69% luminal stenosis) were investigated using a pressure wire; 23 comprised the left anterior descending artery, 9 comprised the left circumflex artery, and 7 comprised the right coronary artery. The ICA diagnosis confirmed a single-, double-, and triple-vessel disease in 14, 11, and 2 cases, respectively. The average of the invasively measured FFR was 0.77 ± 0.13, and 20 vessels (51.3%) were diagnosed with hemodynamically significant CAD (FFR ≤ 0.8). The FFR was measured successfully in all patients.

### Stenosis-related CT-MBF and QPR

Among the CTA assessments of 81 vessels in 27 patients, 32 vessels had no stenosis, 39 vessels had stenoses, and 10 vessels were un-assessable because of calcifications. Significant stenoses in the left anterior descending were observed in 19 vessels, stenoses in the left circumflex were detected in 9 vessels, and stenoses in the right coronary artery were observed in 11 vessels. Of the 49 vessels classified with the CTA-based significant stenosis, 39 vessels were diagnosed with moderate stenosis by the ICA and were measured for the CT-MBF and QPR. Meanwhile, none of the CTA-based non-significant stenotic vessels was diagnosed with the intermediate stenosis in the ICA assessment. The interobserver agreements of the CT-MBF and QPR were 0.89 and 0.85, respectively, and we concluded that the reliability was satisfactory (> 0.70). The CV (95% CI) of the CT-MBF and QPR were 23.1% (17.9–28.4) and 13.4% (10.4–16.5), respectively. There was no overlap in the 95% CIs of the CVs between the CT-MBF and QPR.

**Fig. 2:**
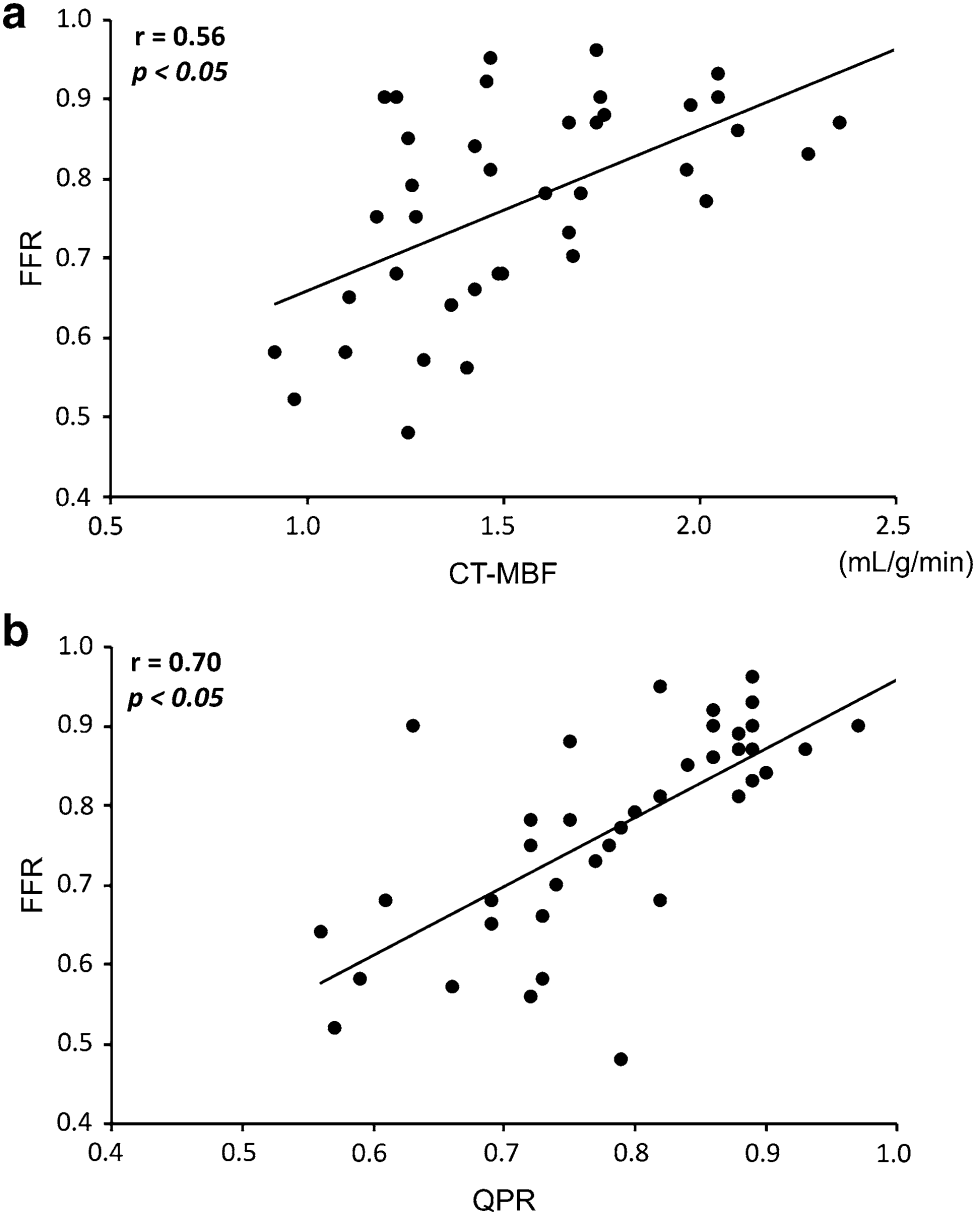
Relationships between the FFR and stenosis-related CT-MBF (**a**) and the QPR (**b**). *CT*-*MBF* computed tomography-derived myocardial blood flow, *QPR* quantitative perfusion ratio, *FFR* fractional flow reserve

**Fig. 3:**
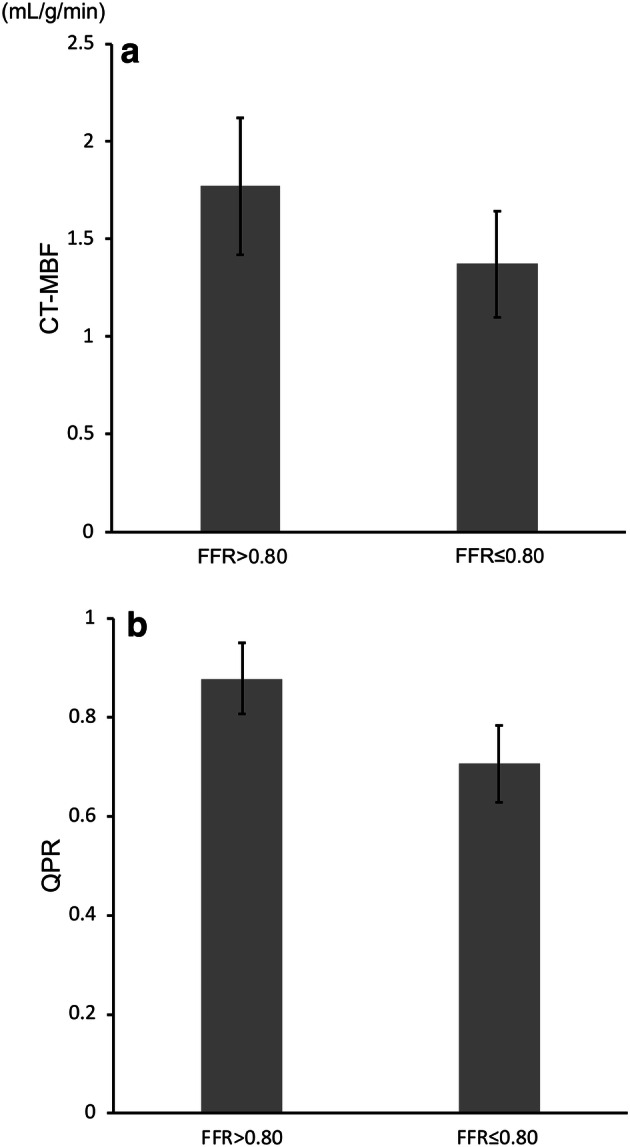
Graphs demonstrating significant differences in the stenosis-related CT-MBF (**a**) and QPR (**b**) between hemodynamically significant and non-significant CAD. Both stenosis-related CT-MBF and QPR in hemodynamically CAD (FFR ≤ 0.8) were significantly lower than those in non-significant CAD (FFR > 0.8) (both *p* < 0.05). *CT*-*MBF*, computed tomography-derived myocardial blood flow, *QPR* quantitative perfusion ratio, *FFR* fractional flow reserve

The overall mean stenosis-related CT-MBF and QPR were 1.55 ± 0.36 mL/g/min and 0.78 ± 0.11, respectively. Significant correlations were seen between the FFR and stenosis-related CT-MBF (*r* = 0.56, *p* < 0.05; Fig. 2a), as well as between the FFR and QPR (*r* = 0.70, *p* < 0.05; Fig. 2b). The stenosis-related CT-MBF in hemodynamically significant CAD (FFR ≤ 0.8) was significantly lower than that in hemodynamically non-significant CAD (FFR > 0.8) (1.38 ± 0.27 mL/g/min vs. 1.74 ± 0.35 mL/g/min, *p* < 0.05; Fig. 3a). Likewise, the stenosis-related CT-QPR in hemodynamically significant CAD (FFR ≤ 0.8) was also significantly lower than that in hemodynamically non-significant CAD (FFR > 0.8) (0.71 ± 0.08 vs. 0.86 ± 0.07, *p* < 0.05; Fig. 3b).

### Diagnostic performance of stenosis-related CT-MBF and QPR

**Fig. 4:**
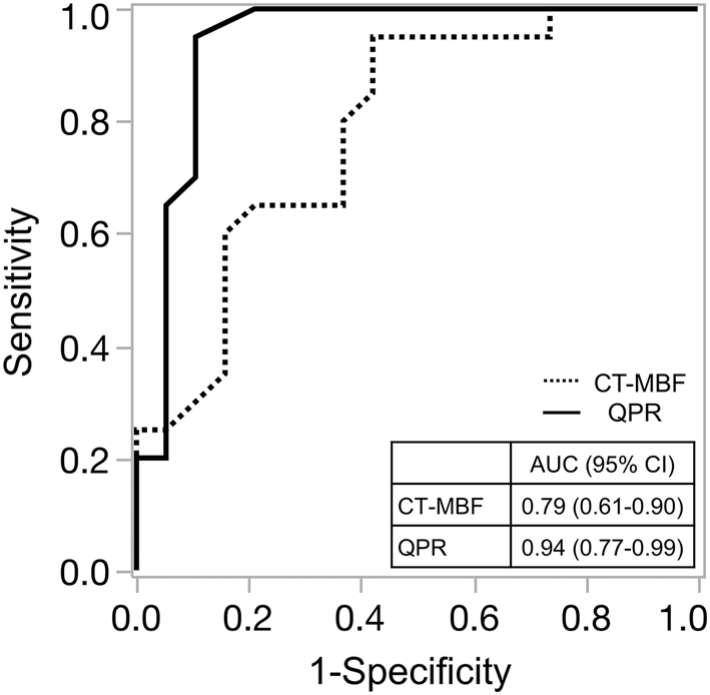
Receiver operating characteristic curves for stenosis-related CT-MBF and QPR to identify hemodynamically significant CAD. *CT*-*MBF* computed tomography-derived myocardial blood flow, *QPR* quantitative perfusion ratio, *CAD* coronary artery disease, *FFR* fractional flow reserve, *AUC* area under the curve, *CI* confidence interval

**Fig. 5:**
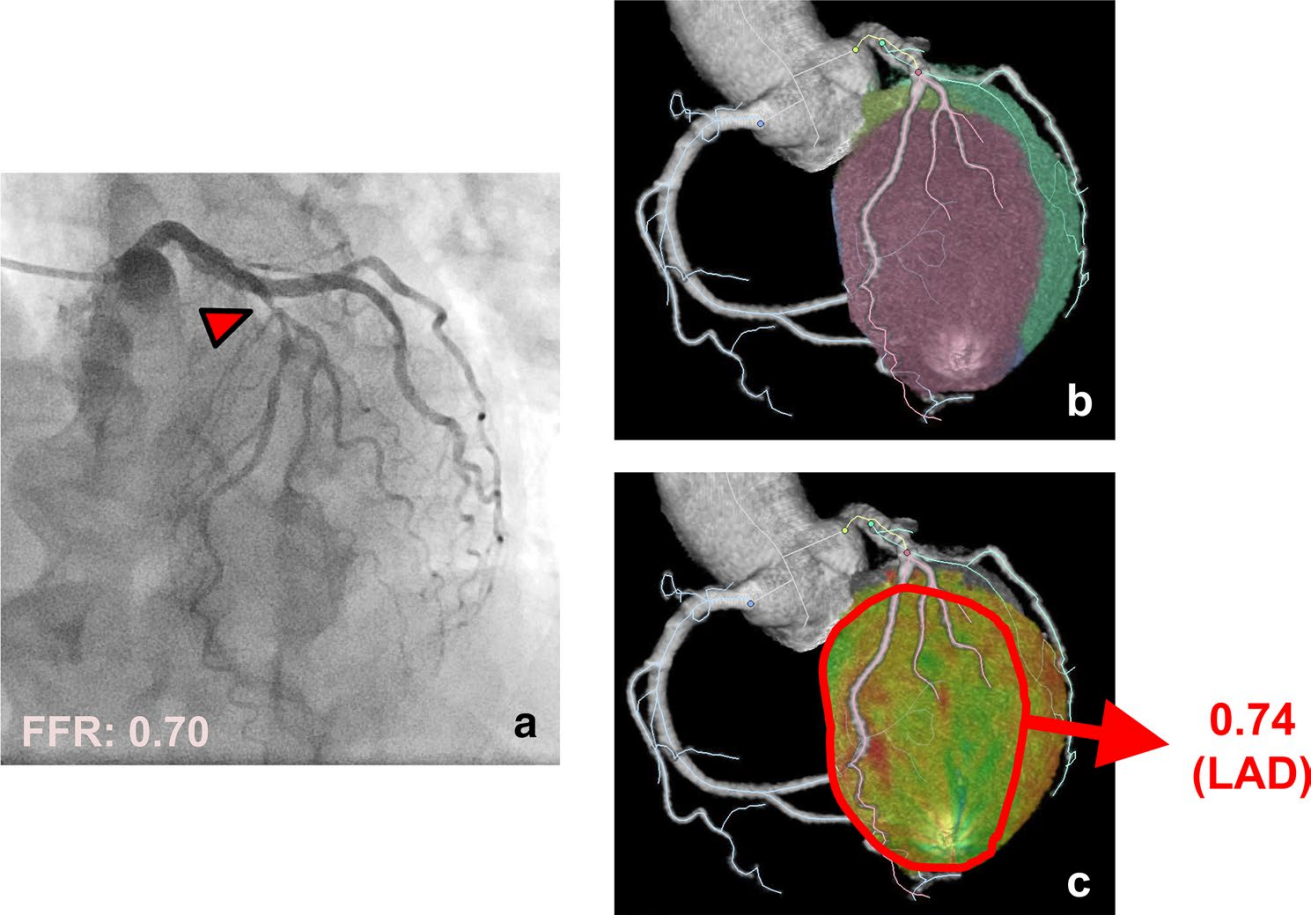
An 80-year-old female with stable angina. The ICA shows significant stenosis in the LAD (**a**). The FFR of this lesion was 0.70. The stenosis-related area was derived using a Voronoi diagram (**b**). The stenosis-related CT-QPR for the lesion was 0.74 (**c**), and the stenosis-related CT-QPR was comparable to the FFR. *ICA* invasive coronary angiography, *LAD* left anterior descending, *FFR* fractional flow reserve, *CT*-*QPR* computed tomography-derived quantitative perfusion ratio

The diagnostic performances of stenosis-related CT-MBF and QPR for detecting hemodynamically significant CAD are shown in Table 2. The cutoff values of the stenosis-related CT-MBF and QPR were 1.70 mL/g/min and 0.8, respectively, according to Youden’s index. The sensitivity and specificity for detecting hemodynamically significant CAD were 95% (95% CI: 85–100) and 58% (95% CI: 36–80) for the stenosis-related CT-MBF, and 95% (95% CI: 85–100) and 90% (95% CI: 76–100) for the stenosis-related CT-QPR, respectively. Figure 4 shows the ROC curves of the stenosis-related CT-MBF and QPR for detecting hemodynamically significant CAD. The AUC of the stenosis-related CT-MBF was 0.79 (95% CI: 0.61–0.90), and that of the stenosis-related CT-QPR was 0.94 (95% CI: 0.77–0.99). The stenosis-related CT-QPR had a significantly higher AUC than the stenosis-related CT-MBF (*p* < 0.05). A representative clinical case is shown in Fig. 5.

**Table 2:** Diagnostic performance of stenosis-related CT-MBF and QPR for detecting hemodynamically significant CAD

	Sensitivity (%)	Specificity (%)	PPV (%)	NPV (%)
Stenosis-related CT-MBF	95 (85–100)	58 (36–80)	70 (61–79)	92 (86–97)
Stenosis-related CT-QPR	95 (85–100)	90 (76–100)	90 (85–96)	94 (90–99)

The data presented in parentheses are the 95% confidence intervals

*CT*-*MBF* computed tomography-derived myocardial blood flow, *CT*-*QPR* computed tomography-derived quantitative perfusion ratio, *CAD* coronary artery disease, *PPV* positive predictive value, *NPV* negative predictive value

## Discussion

The main findings of this study were as follows: (1) The stenosis-related CT-QPR showed a closer correlation with the FFR than the stenosis-related CT-MBF; (2) the stenosis-related CT-QPR had a significantly higher diagnostic performance for detecting hemodynamically significant CAD than the stenosis-related CT-MBF.

A recent meta-analysis showed that the diagnostic performance of myocardial CTP for identifying hemodynamically significant CAD assessed by the FFR was comparable to magnetic resonance imaging or positron emission tomography (PET) and better than single-photon emission tomography [19]. The comprehensive cardiac CT protocol including stress myocardial CTP and coronary CTA allows for the simultaneous provision of morphological information on the coronary artery and functional information on myocardial perfusion in a single assessment. Although the CT-MBF is widely used as a major parameter for the quantitative assessment of myocardial perfusion [7], several studies have indicated that the CT-MBF varies widely depending on the individual, and the optimal cutoff values differ considerably among those studies due to individual factors (e.g., sex, age, coronary risk factors), scan protocol, and differences in the calculation method for CT-MBF [8, 9]. The microvascular function also differs between individuals and influences the CT-MBF value, as well as the FFR [13]. In the present study, the CV of the CT-QPR was significantly smaller than that of the CT-MBF, and the CT-QPR allowed for the correction of the variation in the CT-MBF values. The CT-QPR is less affected by pathophysiological and methodological differences and, therefore, has the potential to more robustly identify hemodynamically significant CAD using a standardized cutoff value. Some recent studies have also demonstrated that relative CT-MBF showed a better diagnostic performance than absolute CT-MBF for assessing hemodynamically significant CAD [20–22]. FFR is based on the principle that the pressure ratio corresponds to the blood flow ratio in the hyperemia state [4]. In our data, the blood flow ratio of the stenosis-related CT-QPR had indeed a strong correlation with the FFR in stress myocardial CTP imaging. Lee et al. also indicated that the relative flow reserve (RFR) had a closer correlation with the FFR than the MBF in ^13^N-ammonia PET [20]. Interestingly, the cutoff values of the stenosis-related CT-QPR and FFR were comparable for detecting hemodynamically significant CAD, indicating that the pressure ratio corresponds to the blood flow ratio in the hyperemia state. On the other hand, the CT-QPR has the potential issue of indicating a false negative in patients with severe three-vessel disease or low left ventricular function because the CT-MBF in the entire myocardium may be decreased leading to a lower reference MBF. However, recent studies showed that stress dynamic CTP can provide unbiased MBF estimates within 20% of rubidium-82 PET that facilitates the detection of multi-vessel disease [23, 24] and, therefore, both the CT-MBF and QPR can be referred to in clinical settings.

Some previous studies have reported that the perfusion territory of coronary artery varies among individuals [25, 26]. Thus, the standard myocardial segment model is not necessarily equal to the individual perfusion territory of each coronary, resulting in a risk for inaccurate assessment of the CT-MBF. We previously reported the feasibility of Voronoi diagram-based myocardial segmentation to assess the perfusion territory of each coronary branch using coronary CTA data [10]. The Voronoi diagram is a mathematical partitioning of a plane into regions on the basis of the distance to points in a specific subset of the plane, and its accuracy has been reported in an animal study [11]. Stenosis-related CT-MBF and QPR with Voronoi diagram-based myocardial segmentation have the potential of more accurately assessing myocardial perfusion of each branch compared to the standard myocardial segment model.

This study has several limitations. First, this study was a retrospective non-randomized study with a relatively small sample size, and it was difficult to further evaluate the impacts of the anatomical location of a coronary artery stenosis on this quantitative index. Second, we evaluated the relationship between the CT-MBF and QPR only for vessels with a stenosis of 30–69% luminal diameter reduction, but not for vessels with less (< 30%) or more (> 70%) severe stenosis without FFR measurement because of clinical discretion. A further study should elucidate the relationship for them as well. Third, the radiation dose was relatively high because this method needs comprehensive cardiac CT examination including dynamic CTP and CTA. If low-tube voltage scan with novel image reconstruction such as iterative model reconstruction is applied to CTA and CTP, further dose reductions can be expected [27, 28]. Moreover, reduction of the sampling rate in dynamic CTP scan is also useful to reduce the radiation dose [29]. However, regarding the clinical use, it is needed to evaluate the value of QPR scanned by the dose-reduced protocol for the further study. Fourth, we did not apply the standardized 17 (16)-segment model for CT-MBF and QPR quantification to clarify the clinical significance of the stenosis-based QPR and CT-MBF for the assessment of individual obstructive CAD. Fifth, the seed points in this study were based on the CTA-based stenosis severity including the un-assessable segments. Coronary CTA can currently predict QCA within ± 25% at best, and the stenosis severity established by QCA does also not always predict hemodynamically significant CAD. Finally, we determined the seed points to CTA-based stenoses without referring to ICA and FFR information for Voronoi diagram-based myocardial segmentation. Further prospective studies are required to evaluate the feasibility of stenosis-related CT-QPR for the prognostic outcomes in patients with CAD.

In conclusion, this pilot study demonstrates that the stenosis-related CT-QPR is a relative hemodynamic quantitative parameter of myocardial perfusion providing accurate information on the perfusion territory of each vessel according to the Voronoi diagram-based myocardial segmentation. The stenosis-related CT-QPR derived from coronary CTA and dynamic CTP imaging showed a significantly higher diagnostic performance than the CT-MBF for detecting hemodynamically significant CAD assessed by the FFR. The clinical usability should be evaluated in the further prospective study with a larger sample size.
